# Compliance of a microstructured, soft sampling device for transcutaneous blood gas monitoring

**DOI:** 10.1039/d0ra03877f

**Published:** 2020-10-05

**Authors:** Ragnar Seton, Greger Thornell, Anders Persson

**Affiliations:** Ångström Space Technology Centre, Div. of Microsystems Technology, Dept. of Materials Science and Engineering, Uppsala University The Ångström Laboratory Lägerhyddsvägen 1, 752 37 Uppsala, Sweden Postal: Box 534 Uppsala 751 21 Sweden ragnar.seton@angstrom.uu.se

## Abstract

Premature neonates are too small for repeated blood sampling, but still require precise monitoring of blood gas levels. The standard method therefore involves transcutaneous blood gas monitoring (TBM), *i.e.* analyzing gas that permeates the skin. The method involves skin heating and requires frequent relocation of a rigid sensor that is adhesively mounted to the skin, which makes the monitoring intermittent and can cause tissue damage. To mitigate this, this paper introduces a TBM concept that replaces the sensor with a small, non-adhesive, flexible, polydimethylsiloxane patch, routing the gases through skin-facing microchannels laid out in various configurations, to an external optical emission spectroscopy system (OES). As the OES depends on a constant flow of gas, we have investigated the effects external loads, both vertical and with a transverse component, have on the aerodynamic resistance of the patches. The experiments show that patches with 200 μm wide channels can withstand uniformly distributed forces up to 25 N with a change in aerodynamic resistance of about 0.01 mbar per sccm per newton. In subsequent measurements, the proof of concept (POC) TBM system showed a strong and fast blood gas signal that was unaffected by all likely loads in the intended application. Moreover, the rise time of the signal is shown to be inversely proportional to the aerodynamic resistance, and the signal strength to be proportional to the skin area exposed to the microchannels. With these results, the POC TBM system is a viable first step towards truly continuous blood gas monitoring of prematurely born children.

## Abstract 2

Replacing rigid transcutaneous blood gas monitoring sensors with microstructured silicone patches, makes the proof of concept system developed and evaluated here a viable first step towards truly continuous measurement on premature neonates.

## Graphical abstract

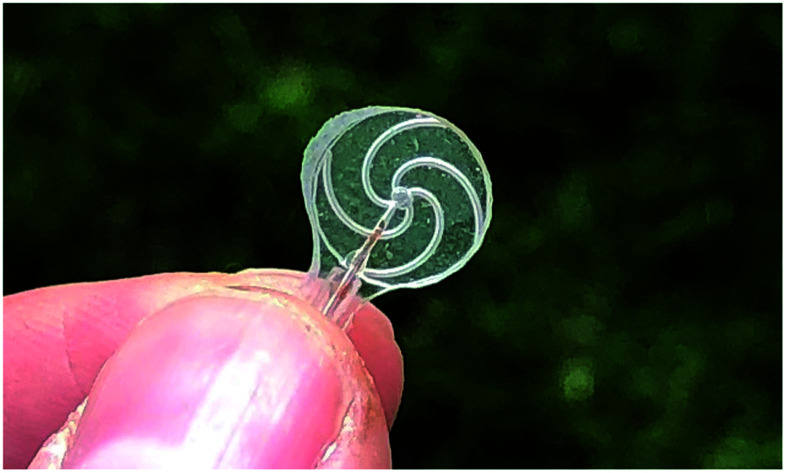

## Introduction

Arterial blood gas measurements of the partial pressure of CO_2_ (*p*CO_2_) and O_2_ (*p*O_2_) provide vital information about the state of the pulmonary function in patients. A large patient group that, due to its elevated risk of pulmonary complications,^1^ often requires continuous monitoring of blood gas levels, is prematurely born infants. Usually, blood gas levels are measured by blood sampling. However, the invasive nature of the test, the drawing of blood, poses several risks for neonates. Besides being painful, it can cause thrombus formations or infections, and for smaller infants in need of continuous monitoring, even necessitate blood transfusions to counter the blood loss.^2,3^

Transcutaneous blood gas monitoring (TBM), on the other hand, is a non-invasive technique developed to be well suited for continuous monitoring, and although it suffers from a need of frequent recalibration and long start up times^3^ it is commonly used. The sensor used in TBM combines the technology of the Clark and Severinghaus electrodes, using a platinum cathode for *p*O_2_ and solid state glass electrode for *p*CO_2_ measurements, respectively.^4^

Modern versions of the sensor have a circular skin surface contact area in the size range of *Ø* ∼ 10 mm and incorporate a heating element to stimulate local hyperaemia^5^ and promote tissue permeability. The typical sensor's housing is just under one cubic centimeter and includes a locking mechanism used with an adhesive pad, very similar to those used for electrocardiography (ECG) electrodes. As preterm neonates have sensitive skin, the standard practice, following the World Health Organization recommendation,^6^ dictates frequent relocation of the sensor to prevent burns and damage to the skin.

With the rigid structure of the sensors, the intrinsic sensor-to-skin surface contact requirement, and the small radii of neonates extremities, the only sufficiently flat, available surfaces are often around the torso of the patients.^7^ This is, however, also the area where ECG electrodes and breathing sensors need to be located.^8^ Hence the possibilities of relocation are constrained, making continuous monitoring exceedingly difficult. In practice, the monitoring becomes intermittent at best. The removal of the aforementioned mounting pads also poses a significant risk of skin damage that has been reported to cause lesions and skin necrosis with severe consequences.^9^

We propose to address these problems by replacing the rigid sensor with a system based on a soft and structurally flexible patch. This will enable attachment to most parts of a neonates' body, including the extremities. Moreover, the proposed system need not be hermetically sealed to the skin, which removes the need for strong adhesives and mounting pads entirely. In the setup described below, the patches have skin-facing microchannels leading the blood gases away from the surface, *via* a capillary into an external optical emission spectrometer (OES) for analysis. The patches are made from polydimethylsiloxane (PDMS), since this silicone based polymer has been shown to facilitate extremely fine features from, *e.g.*, replication,^10^ and also to be well suited for prolonged skin contact.^11^ Furthermore, it has previously been investigated for several medical monitoring applications including soft ECG electrodes^12^ and wearable strain sensors.^13^ While it is sensitive to UV light and certain solvents,^14^ the patches are unlikely to be exposed to harmful doses of either in the intended application.

The full setup provides a proof of concept (POC) that enables measuring blood gases on previously inaccessible parts of a patient's body. However, placing the patches on, *e.g.*, extremities increases the risk of varying external forces acting on it and the compliance of the patches makes them susceptible to deformation under load. In addition, a transverse load could make them lose contact with the skin. As the OES relies on a continuous flow of gas, a deformation could affect the measurement, rendering the system unreliable for unsupervised monitoring. Hence, as a first step towards making the concept an alternative to existing TBM systems, we here investigate and evaluate the fabrication of a set of patches characterized by the geometry of their microchannels, and the force dependence of their aerodynamic resistance, *R*^h^. The latter is evaluated by the effect an applied force has on the aerodynamic resistance of the patch, *i.e.* how the gas flow is affected by deformation. To ensure viability, we also present the results of initial experiments of the complete POC, using the patches together with the OES, as well as correlating these measurements with an existing TBM system.

## Materials and methods

To be able to evaluate (i) the effect external forces acting on a patch has on the gas flow through its channels, and (ii) the influence a patch's channel configuration has on the OES signal, three sets of patches with increasing channel cross section area were fabricated. The cross sections were described by their characteristic width *w*, the width of the channel at skin surface. Each set consisted of two groups of channel configurations, spirals and crosshairs, both with two varying parameters. A patch in the spiral group was uniquely defined by the number of center-to-perimeter channels it has, *N*, and the angle, *Θ*, each channel covers from the center to the perimeter. The crosshairs patches also varied in *N*, but their second parameter, *C*, gave the number of concentric, circular channels they had distributed along their radius. Thus *C* did not, under the long straight channel approximation, *R*^h^ ∝ *L*(*Θ*)*w*^−4^*N*^−1^, where *L*(*Θ*) is the length of the channel, affect the aerodynamic resistance of a patch. All patches could therefore be characterized with respect to *R*^h^ by the vector [*w*, *N*, *Θ*], where *Θ* = 0 for all crosshairs patches.

**Fig. 1:**
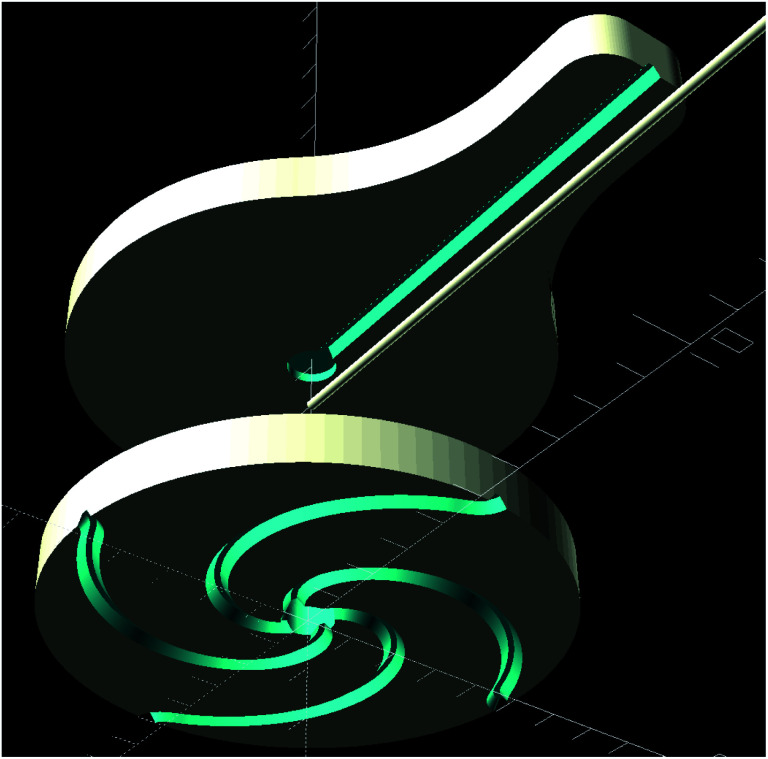
Exploded view showing the top and bottom parts of a patch. The capillary, seen above in the middle, was inserted after bonding and could be retracted and reinserted as needed.

Each patch consisted of two PDMS parts, see Fig. 1, that were bonded together. The bottom part faced the skin and contained the above mentioned channel configurations, whereas the top part only had a single channel for capillary insertion, connecting the patch to the OES system.

**Fig. 2:**
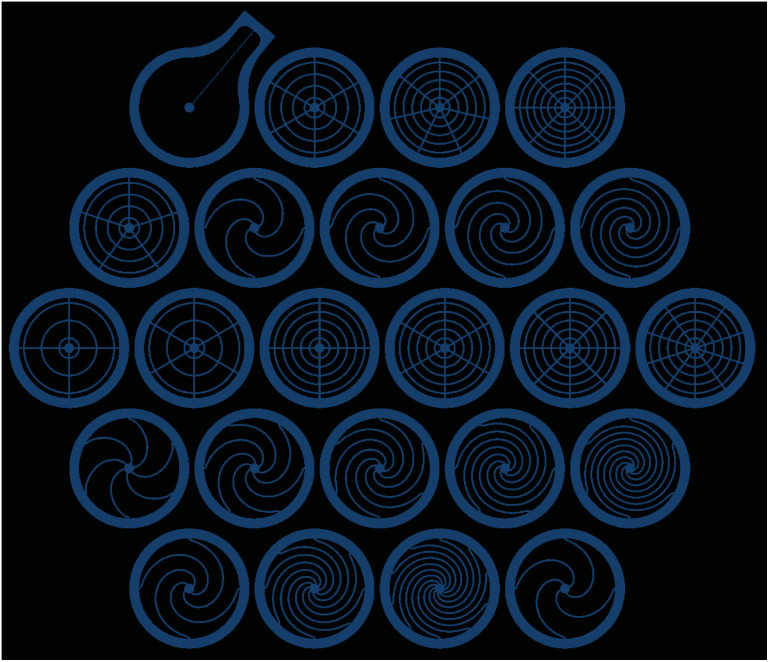
Bottom part patch master milling paths containing all configurations used in the experiments. Note that the first and last configurations in the bottom row are the same as the third and second one from the left in second row from the top. This positive master was milled in a CNC router, with the wider paths shown above milled deeper such that the negative master being cast in it would have walls within which the final patch bottoms could be cast. The inner radius of the walls, *i.e.* the radius of each patch, was 5 mm and the radius of the center circle, forming the hole through which gas would flow into the capillary, was 0.5 mm.

Whereas the three sets of patch bottoms, defined by their shared *w* ∈{146, 200, 400} μm, were comprised by 21 unique configurations given in Table 1, the top parts were all identical with a capillary channel width of 200 μm. Hence the casting of the top parts only required a single master while the bottoms required one for each value of *w*. In Fig. 2 one of these bottom masters is depicted, as seen in the top left corner it also includes a single top. Since the bottom masters were fabricated before the top ones, one top was created for each *w*, and could thus be tested before deciding on a *w* for all tops.

**Table 1:** Channel configurations for patch bottom sets. Each column in the two bottom rows represents a subset of the patches with the same number of channels, *N*. This follows from *Θ* ≠ 0 ⇒ *C* = 0 and *C* ≠ 0 ⇒ *Θ* = 0, and gives a total of 21 unique configurations

*N*	4	5	6	7	8	10
*Θ*	180°, 270°, 360°, 450°	—	90°, 180°, 270°, 360°, 450°	—	270°	270°
*C*	3, 5	4	3, 4, 5	5	5, 6	5

### Patch fabrication

The process of fabricating the top and bottom parts involved two molds: a positive, solid master which did not deteriorate from use, and a negative PDMS mold, which, while not as robust as the former, could be reused several times. The positive one was milled in a glass reinforced epoxy laminate (FR4 without copper plating) using a CNC router (ProtoMat S104, LPKF, Germany). After rinsing and drying it thoroughly, it was coated with perfluorooctyltriethoxysilane (Silane 667420, Sigma-Aldrich, USA) by passive evaporation deposition, *i.e.* placing the mold in a vacuum desiccator (Nalgene 5311-0250, Thermo Fisher Scientific, USA), pumping (Laboport N 840.1.2 FT.18, KNF Neuberger GmbH, Germany) it to a low, >90 mbar, vacuum and letting a 10 ml Silane drop evaporate and deposit at 21 °C for 12 hours. The Silane was required to ensure that the high-aspect-ratio features of the negative mold would not break when separating the two.

The aforementioned negative mold was cast by depositing PDMS (ELASTOSIL RT 601, Wacker, Germany), held in place by surface tension, on top of the positive mold, followed by curing at 95 °C for two hours. In order to make the PDMS penetrate the finer and deeper features, trapped air was removed using a syringe with a 32-gauge (250 μm) needle. After separating the molds, the negative one was treated in a 40 kHz, 200 W, air plasma oven (Atto-BL-PCCE, Diener Electronic, Germany) for 60 s to generate a surface release agent, as described by Shao *et al.*,^15^ before finally casting the patches and letting them cure at 21 °C for 12 hours. Curing them at a higher temperature had an adverse effect on the surface release agent, resulting in a very low yield of patches and often destroying the mold as well, during the separation.

The fabrication stability in terms of mold size consistency was evaluated using an optical profiler (Nexview, Zygo, USA) after each replication.

### Bonding the patches

The general process of bonding PDMS to PDMS is well documented.^16^ However, as the number of types and manufacturers have increased, studies of optimizing it^17^ has also become more type specific. To reach sufficient bonding strength a process was therefore developed, and while based on the work of Duffy *et al.*,^16^ it contained a series of steps not described therein.

The process started with cleaning the surfaces from small particles using Scotch tape (3M), then rinsing them using isopropanol (IPA) and deionized water. This was followed by a 60 second, 500 kHz, 5 W corona treatment using a BD-50E Heavy Duty Generator (Electro-Technic Products, USA), where the T-shaped tip was swept back and forth over the surfaces at a distance of 10 mm, before swiftly placing them on top of each other and applying a constant pressure of 5.4 N, at 90 °C for 12 hours. The timespan from corona treatment to surface contact was found to be of great importance, and that exceeding 30 s had a very detrimental effect on the bonding strength. This, together with the width of the corona tip, were the two main limiting factors for the bonding batch sizes.

**Fig. 3:**
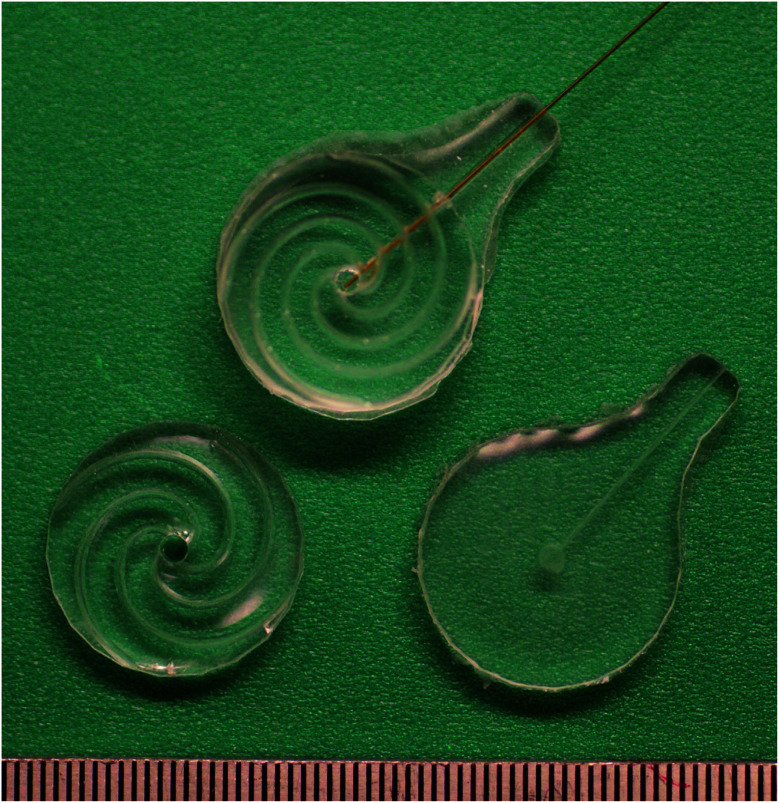
Top and bottom parts, together with a bonded patch showing minimal wear post several force experiments. 0.5 mm ruler for scale, and a capillary with an outer radius of 150 μm inserted.

During the process, only the center holes were ensured to be aligned, see Fig. 3. Hence, there was no correlation between the top's capillary channel and the bottom's channel configuration.

### Patch evaluation

**Fig. 4:**
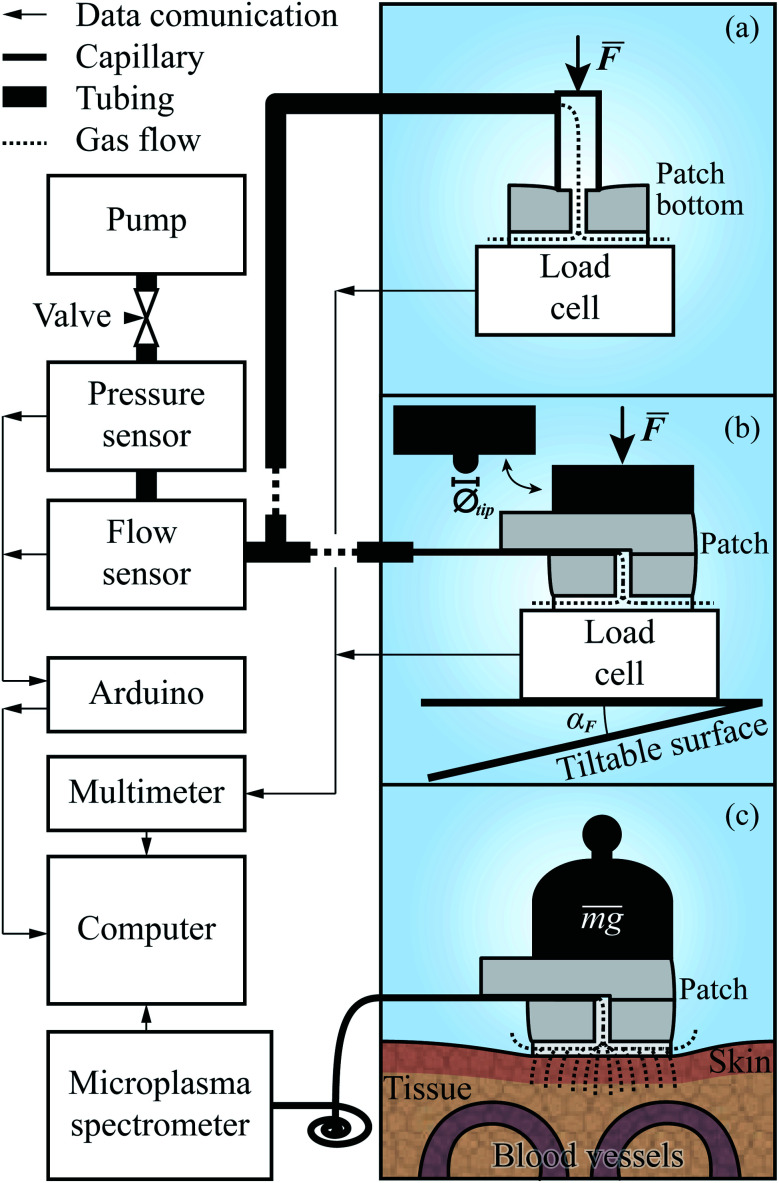
Experimental setup for patch evaluation and TBM measurements.

The setup for evaluating the *R*^h^(*F*) of the patches, schematically depicted in Fig. 4a, included two separate data acquisition units, a Keysight (formerly Agilent, USA) 34972A, to which a single point bending beam load cell (0–50 N, made in house) was connected, and an Arduino UNO R3 connected to the flow sensor (AWM3100V, Honeywell, USA) and pressure sensor (AMS-5915, Analog Microelectronics, Germany). The synchronization of the measurements was performed on the computer using a MATLAB script which took into account the known latencies in the system.

The first experiment, measuring the aerodynamic resistance of the patches, took place before the patches were bonded together. Before each measurement, a patch bottom was placed on a clean mirror surface on top of the load cell, mounted on a vertically adjustable, and tiltable, platform. Above the load cell, and directly connected to the measurement system, a beveled pipe with a 3.5 mm inner radius, 2.5 mm through wall thickness, and 15° bevel, was mounted, through which the air would flow.

A measurement series was initiated by starting the pump (EDM6, Edwards Vacuum, UK) and adjusting the needle valve to ensure that the flow stayed within the range of the flow sensor. Following this, the patch bottom was moved up into contact with the pipe. By further pressing them together, the pipe end–patch interface was sealed, and as a steady state of compression was reached, the flow and pressure were measured. This procedure was repeated for all patches before preparing the setup for the subsequent experiments by replacing the pipe with a longer, flexible tube with a 37.5 mm long, fused silica capillary (TSP075375, Genetec, Sweden) ending. The capillary had an inner radius of 75 μm, and an outer one of 375 μm. The former was matched with the length of the capillary to ensure that the aerodynamic resistance of the system following the patch, to the left of it in Fig. 4b, would be less than, or in the same order as, that of the microchannels. The latter was large enough to seal the interface between the capillary and the *w* = 200 μm channel in the top part of the patch while still being retractable. The next preparatory steps were to mount an aluminum rod with a 6.75 mm radius above the platform and then bond the top and bottom parts of the patches together.

The second experiment was initiated by inserting the capillary into a patch, as depicted in Fig. 3, and placing it in the center of the platform. It was then raised, causing the rod to apply an uniformly distributed force over the patch. As in the first experiment, the force, pressure, and flow rate were recorded, and after the *R*^h^ of the compressed patch had reached a steady state under the peak force, the platform was lowered again, and the patch was replaced. For the subsequent experiments, the sequence of the measurements was the same. The difference in execution was for the third experiment that the force was applied at an angle, *α*_F_ ∈ {1°, 2°, 4°}, acquired by tilting the platform as shown at the bottom of Fig. 4b. In the fourth, the platform was again orthogonal to the rod. However, on the rod's patch-facing end, a tip with a radius of 1.175 mm (*Ø*_tip_ = 2.35 mm) was placed, through which a point force was applied. The experiment included measurements with the force applied at two different points, at distances *p*_r_ ∈{*r*_patch_/2, *r*_patch_} behind the center of the patch, along the line given by the capillary channel.

In the third and fourth experiment, the respective initial, and only point of contact between the patch and rod were considered random with respect to the channel configuration of the patch. The number of patches was sufficient to cover critical cases such as an angled force's initial point of contact being directly on a single channel exit, and a point force applied directly onto a channel.

### System evaluation

The transcutaneous measurements were performed by connecting the patches to the OES system *via* a single, 190 mm long capillary with an inner radius of 40 μm. The dimensions of the capillary ensured that the aerodynamic resistance of the connection to the OES system would be significantly higher than that of the patches themselves, contrary to the previous experiments.

In the OES system, the sampled gas flowed through a microplasma source at a flow rate of 0.1 μmol s^−1^ and a pressure of 100 Pa. The plasma source was a stripline split-ring resonator^18^ with a 2 mm wide plasma gap, inside which the sample was partly ionized in a 2.5 GHz microwave electric field. Upon ionization, the sample emitted species-specific radiation that was analyzed to determine its CO_2_ content. This analysis was performed with a CCD spectrometer (CCS200, Thorlabs, USA) with a bandwidth of 200–1000 nm, a FWHM of 2 nm at 633 nm, a 20 μm by 2 mm slit, and a 600 lines per mm, 800 nm Blaze wavelength grating. The complete system is thoroughly described by Persson and Berglund.^19^

Before a measurement began, the OES system was initialized and allowed to stabilize for >30 min. Then, the capillary was inserted into the patch, after which the OES system was allowed to stabilize for another minute, before the patch was attached to one of the authors' lower arm with a piece of tape. The purpose of the tape was solely to hold the patch in place and facilitate rapid replacements. It exerted a negligible force onto it. In a potential, future product, the compliancy of the patch will make it possible to use a simple dressing to avoid the aforementioned problems related to adhesives.

The measurement site was chosen to avoid any hair, and was properly washed with soap before the experiment. The site boundary was marked by pen to ensure that the same site was used for all measurements. During measurements, the arm was resting on a table with the elbow in a 90° angle. After the patch was attached to the site, the signal was first allowed to stabilize to record its rise time, then its strength was estimated by averaging it for about 120 seconds, corresponding to around 150 samples with an average sampling frequency of 1.24 Hz. The patch was subsequently removed, and the signal fall time recorded. When the signal had returned to the starting level, the capillary was retracted from the patch and inserted into a new one, restarting the measurement sequence. To test how the TBM measurements were affected by the deformation of the patches, cylindrical 100 g weights were loaded onto the patch while it was attached to the skin. In this experiment, the patch was again attached horizontally on the arm and the weights were lowered through a vertically mounted pipe with a 0.5 mm larger radius to avoid that the force was applied at an angle. The TBM signal was then recorded while adding a new weight about every minute until the total mass was 300 g, which, consequently, corresponded to a nominal force of about 3 N and a pressure of 3.8 kPa.

The data was recorded and analyzed by the same software used by Persson and Berglund,^19^ where the CO_2_ content of the sample was estimated by studying the 0–3 band of the CO Ångström system. The intensity of this emission band – centered at 560 nm – was linearly dependent on the CO_2_ content.^19^ The spectroscopic signal was extracted in three steps where, (i) the spectrum was filtered with respect to both wavelength and time with smoothing average filters of power 2 and 7, respectively, (ii) the background was subtracted by fitting a four-term Gaussian model to the spectrum in the wavelength interval 552–564 nm, but excluding points between 558 and 562 nm, *i.e.* the CO_2_-dependent interval, and (iii) the spectroscopic signal was then defined as the integral of the latter interval.

To calculate the strength of the transcutaneous response, a first-degree polynomial was fitted to the ten first and ten last points of the spectroscopic measurement, *i.e.* the parts of the measurement where the patch was not attached to the skin. The signal was then scaled by this polynomial to estimate the relative signal change when the patch was attached. The strength, *S*_T_, and stability, *σ*_T_, of the transcutaneous signal were calculated as the average and standard deviation of the stable part of the measurements. Finally, the rise and fall times of the signal, *t*_R_ and *t*_F_, were calculated as the times it took the signal to go from 10% to 90% and 90% to 10% of *S*_T_, respectively.

In addition to the experiments studying the influence of the patch geometry, and its deformation, on the transcutaneous signal, a reference experiment with a commercial TBM system (OxiVenT, SenTec, Switzerland) was also conducted. Here, both systems were attached to the subject's arm and used simultaneously to study the change in transcutaneous CO_2_ pressure created by occluding the arm above the elbow with a blood pressure cuff.

## Results and discussion

**Fig. 5:**
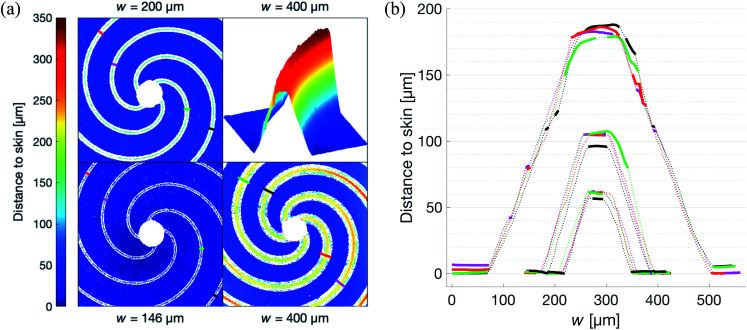
Optical profiling results showing the topographical and cross sectional profiles of patches from each set. (a) Topographical profile of the patches. Plotted lines correspond to the cross section profile with the same color in (b). Top right quadrant shows a channel segment with interpolated channel walls. (b) Cross sectional profiles of the patches shown in (a). Dotted lines indicate nearest neighbor interpolation values in the surface, as illustrated by the top right quadrant in (a), solid are measured.

The optical profiler measurements of the patches show that the lateral precision in the fabrication process is limited by that of the CNC router. The casting did not cause any measurable differences compared to the master, keeping the standard deviation in *w* below 2 μm as shown by Fig. 5b. The roughness of the vertically propagating features in the patches were attributed to the positive FR4 master. As seen in Fig. 5a, these defects are primarily concentrated to the narrow bottom of the channel where the effect on the flow is limited. It is worth noting that one of the primary reasons for choosing FR4 as material for the master was its compatibility with the tools available in our laboratory. In a possible production setting, the issue of these defects could be addressed by choosing a more cohesive material such as aluminium.

**Fig. 6:**
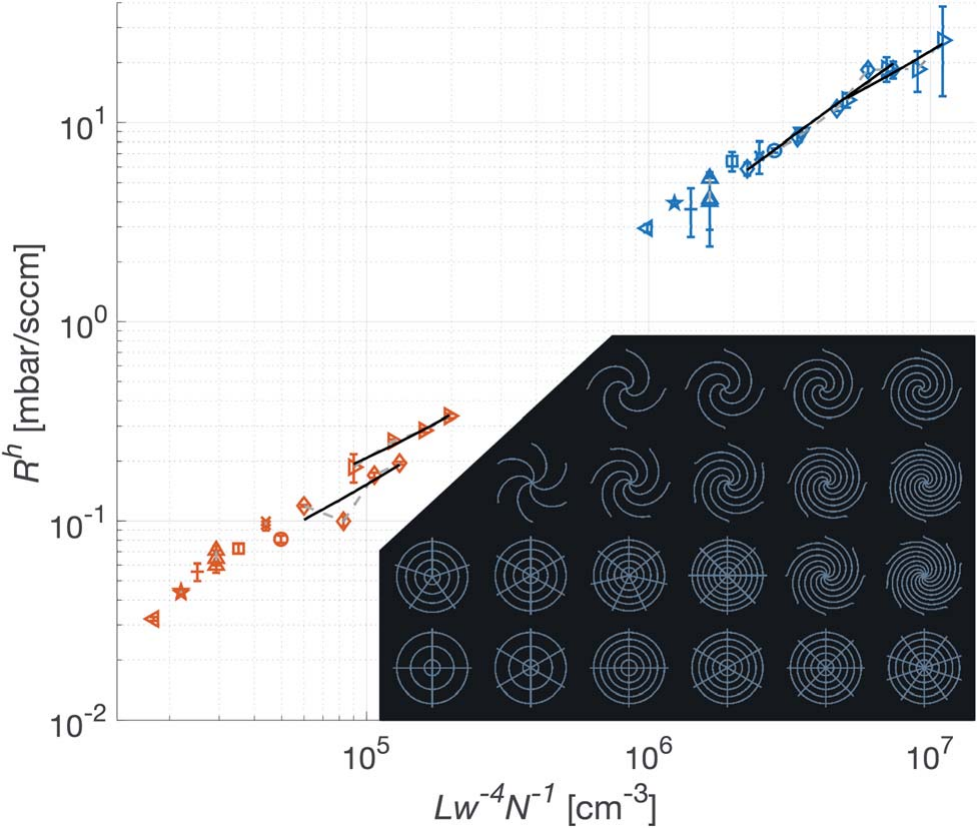
Verification of the validity of the theoretical framework, and stability of the fabrication process. The measurements were performed on only the skin-facing, bottom part of the patches. The red markers correspond to patches with *w* = 400 μm and blue *w* = 146 μm. The solid black lines are linear regressions of patches with the same *Θ* and increasing *N* (connected with gray dashed lines). Inset in the graph are all the measured patch configurations.

The low intra-set variability in *w* is affirmed by the validation of the approximation of translational invariance along the axis of propagation of the gas, *i.e.* the long straight channel approximation. As shown in Fig. 6, this holds for all patches. In this figure, where only the bottom part of the patches are used, the *R*^h^ is indeed shown to be proportional to *Lw*^−4^*N*^−1^, where *L* is measured from the edge of the center hole, along the center of the channel, to the perimeter of the patch. Additionally, the concentric channels in the crosshairs patches are confirmed to have no measurable effect on the *R*^h^.

For the spiral patches, experimental verification of this approximation was of importance as, in some configurations, the magnitude of the radius of the curvature of the channels approached the length of them. Hence it was not known to what extent the Hagen–Poiseuille description, as derived by Landau and Lifshitz,^20^ could be applied.

### Force dependence of flow resistance

**Fig. 7:**
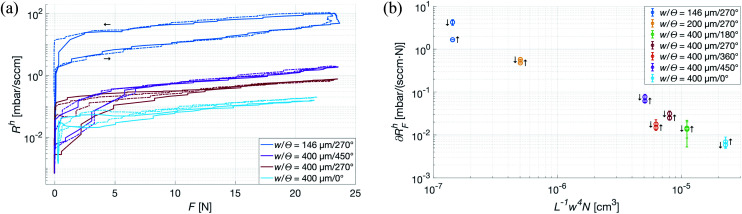
Evaluation of the force dependence of the aerodynamic resistance of all bonded patches. (a) Raw signal from two measurements (solid and dash-dotted line) of uniform force, over a selected group of *N* = 4 patches. Rising and falling force are for the [*w* = 146 μm, *N* = 4, *Θ* = 270°] curves indicated by → and ←, respectively. (b) Force dependence of all bonded patches calculated for both rising and falling edges, indicated in the figure by ↑ and ↓, respectively. As seen, the diminishing effect of an applied force, while primarily *w*-dependent, is proportional to (*R*^h^)^−1^.

With the theoretical model for gas flow in the channels verified, the next step was to determine the impact different types of deformation of the patches would have on the flow. In the subsequent experiment, the deformation-inducing force was applied uniformly over the top of the patch along its normal, restricting the deformation to the (macroscopic) plane. As seen in Fig. 7a, the maximum applied force is the equivalent of placing a 500 kg weight on a 20 × 13 cm hand. This upper limit is of course far beyond anything a patch is likely to be subjected to if used in an healthcare application, even on adult patients. However, it shows that the log-linear force dependence, ∂*R*^h^_F_, of the aerodynamic resistance is consistent throughout the experiment – which in turn gives the possibility to further optimize the design of the patches. As the system relies on the patches having a constant, time-averaged, flow of gas through its channels, it was important to not only determine the ∂*R*^h^_F_ but also how the parameters of the patches influence it. Analogous to electric conductivity, Fig. 7b shows that while ∂*R*^h^_F_ under a uniform, vertical force primarily depends on the characteristic width of the channels, it does decrease with (*R*^h^)^−1^. This is a somewhat less than intuitive result, as *N* will of course be unaffected by deformation, and the relative changes in *L* were expected to be negligible in comparison to those in *w*. Nevertheless, these results show that uniformly distributed forces, such as those exerted by a dressing mount of the patches on a neonate or even a strapped mount on an adult patient, have a negligible effect on the flow through channels with a large *w*.

The significant discrepancy between ∂*R*^h^_F_ on the rising and falling edges of the force curve is explained by a slight drift in the pressure sensor at very low pressures. Fig. 7b shows ∂*R*^h^_F_ for both edges, however only the rising one was used in further analysis.

**Fig. 8:**
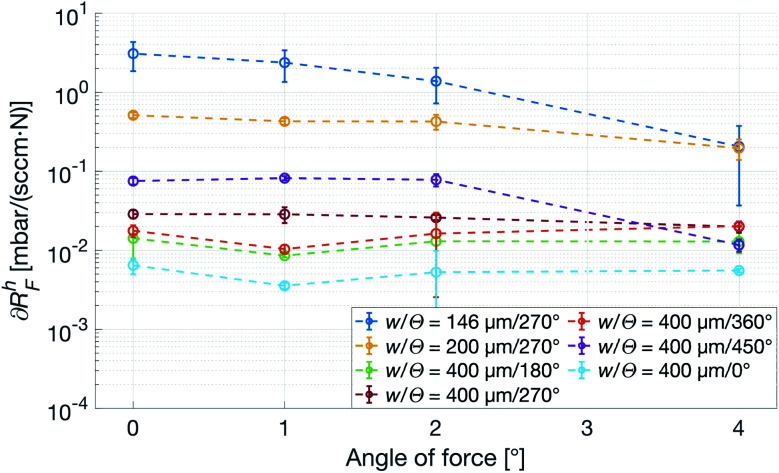
Force dependence of the *R*^h^ with respect to angle of incidence of the force, *α*_F_, calculated on the rising edge of the force curve. All patches have *N* = 4.

To mimic a load more similar to one that would occur when a patient touches or presses the patch against a surface, a force with a transverse component acting on the top of the patch was used. The deformation in this case is no longer restricted, and especially the patches with a smaller *w* are observed to tilt slightly, losing contact with the counter surface and thus sharply reducing its *R*^h^. As seen in Fig. 8, ∂*R*^h^_F_ of the most narrow channels are dropping close to a magnitude, whereas the wider ones show no angular dependence, indicating that *w* has the previously expected dominant influence. Also, from the small-*w* curves in Fig. 8, it is clear that an increasing transverse component of the force corresponds to a decrease in force dependence of *R*^h^.

**Fig. 9:**
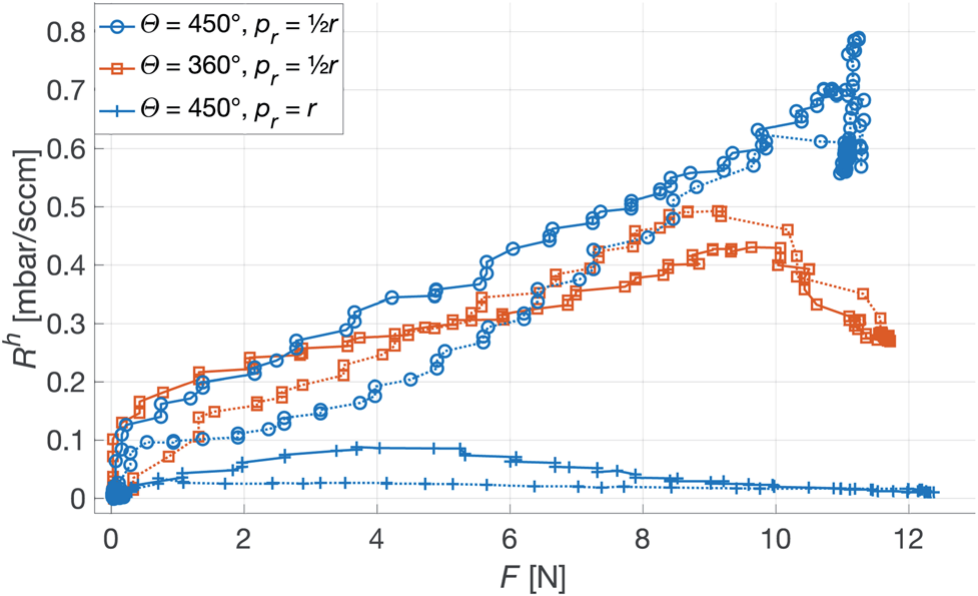
Raw signal of point force over selected [*w* = 400 μm, *N* = 4] patches. Solid and dotted lines indicate rising and falling forces, respectively. The load causes three different types of deformation: complete channel blocking (top curve), patch folding (bottom curve), and channel blocking followed by folding (middle curve).

In the experiment characterizing the effects of pointwise deformation, it became clear that the system was sensitive to this kind of force. The initial experiment included measurements for three points on each patch, *p*_r_ ∈{0, *r*_patch_/2, *r*_patch_}. However, *p*_r_ = 0 repeatedly caused the patches to crack, rendering the measurements unusable, and was therefore omitted. In all the subsequent measurements, the patches would deform to either, (i) have a channel collapse causing a steep increase in the *R*^h^, (ii) quickly fold up around the point of pressure causing the *R*^h^ to drop to 0, (iii) a combination of the two. The three regimes are illustrated by the results of the three measurements shown in Fig. 9. While this presents a failure mode of the system, we deem the probability of such a load in the intended application to be low. Moreover, as the changes in *R*^h^ are fast and significant, rather than small and gradual, it is unlikely that the corresponding change in output signal from the OES is interpreted as a change in *p*CO_2_ or *p*O_2_, but rather as the patch failure it is.

### Transcutaneous blood gas measurement

**Fig. 10:**
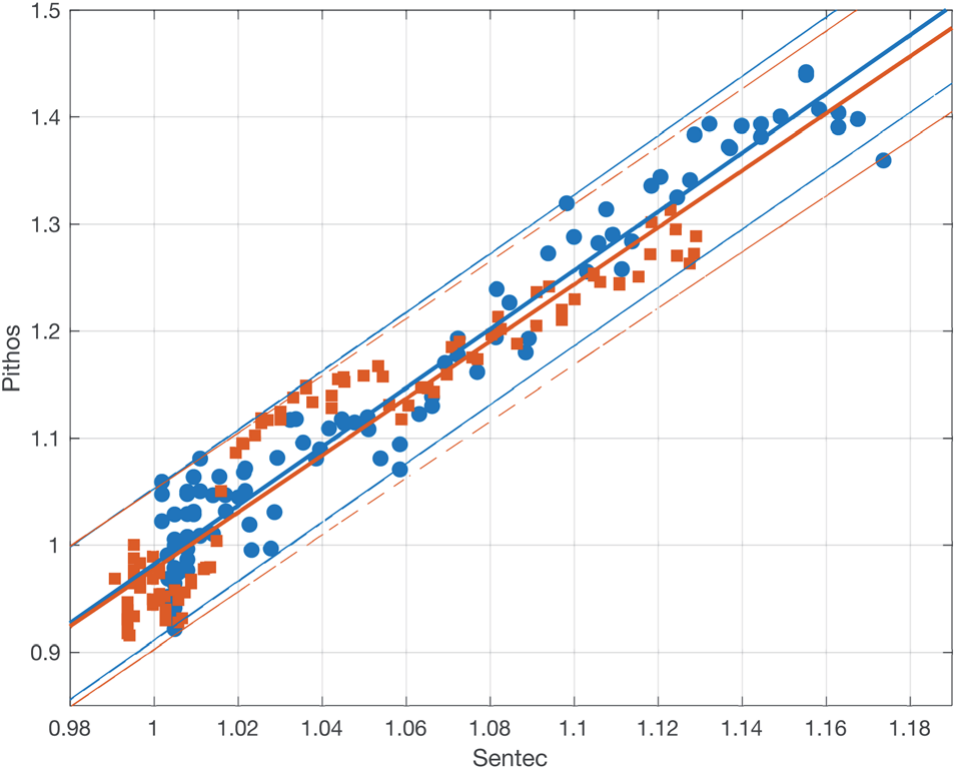
Correlation between the CO_2_ signals from the OES and commercial TBM systems in two experiments (red squares and blue circles) with the latter on the *x* axis and the former system on the *y* axis. The signals were normalized by the average signal before the occlusion. The solid and dashed lines show linear fits and 95% prediction bounds to the two experiments. The average slope of the fits is 2.7.

Fig. 10 shows the comparison between the OES system and the commercial TBM system. The figure shows the relative change in the signals after the capillary CO_2_ pressure started to rise due to the occlusion of the arm in two repeated experiments. As can be seen, the two signals showed a linear correlation with good reproducibility, indicating that the OES system delivered reliable transcutaneous measurements. It can also be mentioned that in these experiments the present system showed almost three times higher sensitivity than the commercial device.

**Fig. 11:**
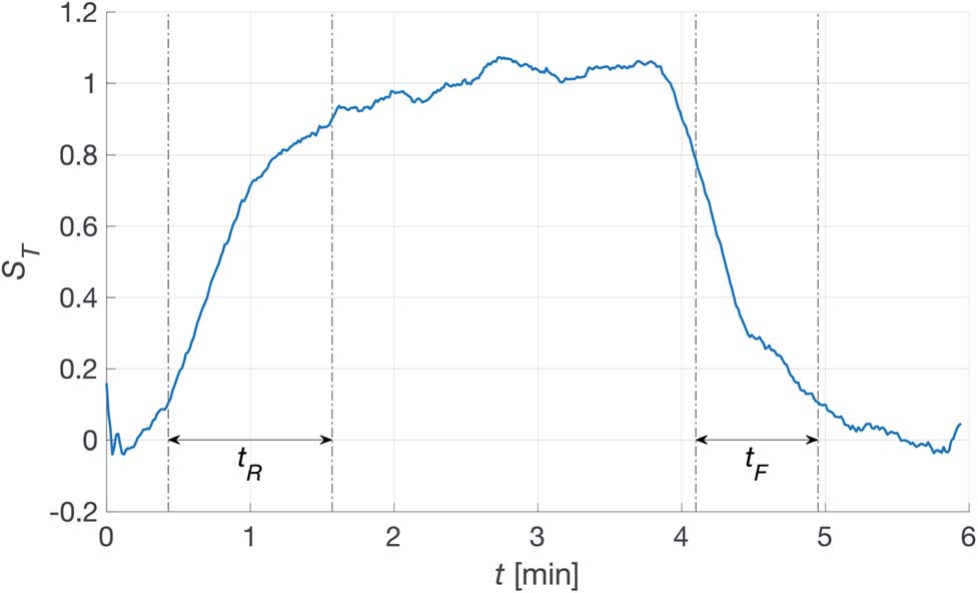
Transcutaneous signal from the [*w* = 400 μm, *N* = 4, *Θ* = 270°] patch. The patch was attached to the skin at *t* ∼ 0.2 min and removed at *t* ∼ 3.8 min. The dash-dotted lines show the intervals where the rise and fall times were calculated. The signal strength and standard deviation were calculated in between the two.

**Fig. 12:**
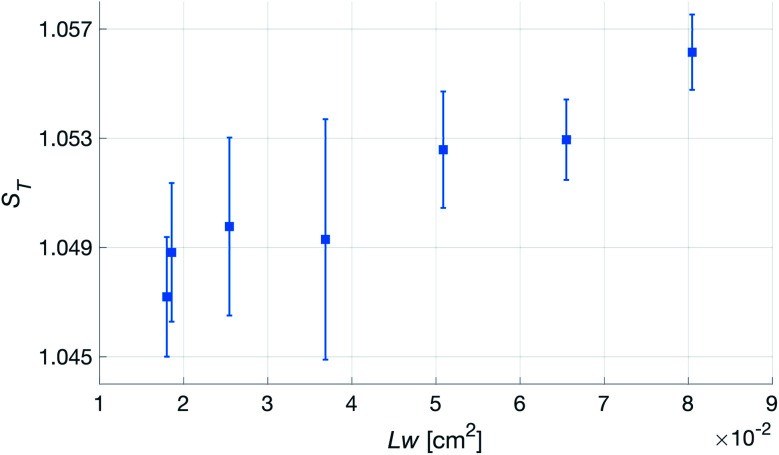
Transcutaneous signal strength, *S*_T_, as a function of skin area exposed by the channels, *Lw*, for patches with *N* = 4.

**Fig. 13:**
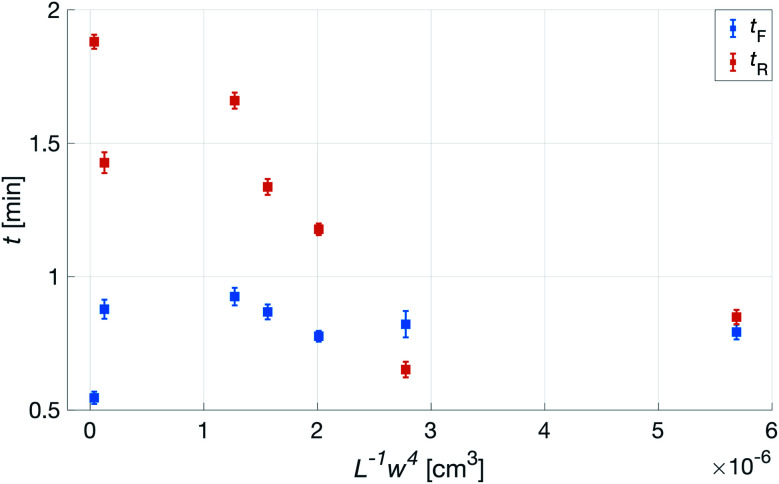
Transcutaneous signal rise, *t*_R_, and fall, *t*_F_, times as a function of *L*^−1^*w*^4^ for patches with *N* = 4. *L*^−1^*w*^4^ being inversely proportional to *R*^h^.

**Fig. 14:**
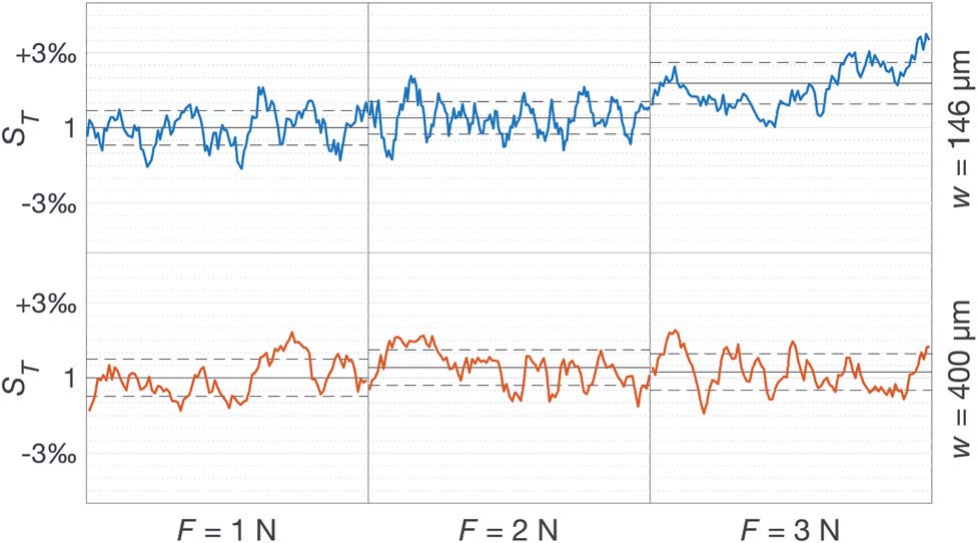
Change in transcutaneous signal during compression with forces between 1 and 3 N of a [*w* = 146 μm, *N* = 4, *Θ* = 270°] and a [*w* = 400 μm, *N* = 4, *Θ* = 270°] patch. The solid black line corresponds to the average over one minute, and the dashed lines to the standard deviation of each measurement. All values are normalized to the average of the *F* = 1 N measurement on each patch.

Having established that the *R*^h^ was sensitive to deformation, and that this sensitivity depended on the geometry of the channels, it was important to investigate how both the patch geometry and deformation affected the transcutaneous blood gas measurement. Especially since the plasma source of the spectrometer had previously been shown to be susceptible to small changes in pressure and flow.^21^Fig. 11 shows a typical transcutaneous CO_2_ measurement result, from which the rise and fall times of the signal are obtained along with its strength. From an application point of view, a transcutaneous blood gas monitor should have a strong and stable signal with a short rise time. Hence, if these properties were associated with patches with small *w*, which this study shows are the ones most sensitive to deformation, the proposed concept would have a problem. Fortunately, Fig. 12 shows that both the strength and the stability of the signal are dependent on the area of skin exposed by the channels, *Lw*, rather than just the characteristic width, *w*, or *R*^h^. This is reasonable, since this interface is the principal path for the blood gases to enter the system, and fortunate, since the best performance is achieved with patches having large *w*, low *R*^h^ and little susceptibility to deformation. Moreover, Fig. 13 shows that the rise time of the signal scales inversely with *R*^h^. For the two investigated patches with the lowest *R*^h^, [*w* = 400 μm, *N* = 4, *Θ* = 0° and 180°], the rise time is of the same order as the fall time, during which the patch is not attached to the skin, meaning that the channel structure does not contribute to the effect at all. However, despite the indication that the patches that are least susceptible to deformation also produce the best transcutaneous measurements, the risk that even these small deformations could affect the signal remained. Hence, in the final experiment, patches with large and small characteristic widths were subjected to deformation by compression, with forces between 1 and 3 N while being attached to the skin. The results, Fig. 14, show that the signal from the patch with the smaller *w* is significantly affected by compression at forces between 2 and 3 N, while the signal from the patch with the larger *w* remains stable throughout the investigated force interval.

Combined, these results show that it is possible to create a transcutaneous blood gas monitor with a flexible, and thus gentle skin interface that is resilient to deformation in any range relevant to the intended healthcare applications. They also indicate that the OES system can offer other advantages such as greatly improved sensitivity. However, there is still much to be learned about the underlying mechanisms of this soft system, *e.g.* what the limits for gas concentration monitoring are, and how they compare to the transcutaneous gas flow on different patients.

## Conclusions

We have here designed a POC system for TBM, primarily targeting prematurely born neonates, fabricated and evaluated the passive patches used in it and performed initial viability experiments of the complete system. The combination of double casting PDMS to fabricate the patches, and having a long term reusable positive master milled in a CNC router, enables both fast single-shot fabrication, and long term stability.

By moving the gas analysis to an external OES and utilizing these small, flexible patches, some of the inherent limitations associated with the traditional sensors have been avoided. The low contact force needed, makes adhesive mounting redundant and the compliance makes it possible to place them on small, rounded surfaces such as the extremities of a neonate. The sensitivity analysis of the force dependence of the aerodynamic resistance of the patches shows that point forces are able to deform the patches to an extent where gas will no longer flow through the microchannels. However, for our intended application, this is an unlikely scenario. The more likely case of a uniformly distributed force over the full patch will, given a sufficiently large width of the channels, if at all, only affect the resistance in a small and highly predictable way, making the patches well suited for use with the OES. Our experiments also show that the top part of the patches serve as sufficient protection for the capillary, as no breaking occurred, meaning we can at this point see no reason to introduce non-flexible parts in this soft system. Furthermore, the wide range of loads investigated here shows that there is ample opportunity to further optimize the design of the patch, as the load placed on the patch does not seem to be a limiting factor. The TBM experiments showed short rise and fall times, high SNR, and confirmed the small uniform force dependence of aerodynamic resistance of patches with large characteristic widths. In the comparison with an established TBM device, our POC system outperformed it in several respects, *e.g.* sensitivity. Although this is not a conclusive comparative study, it shows that it is a concept worth developing further. Based on this, we believe that, while further investigation of the system is required, the concept shows great promise and could in the future serve as an option in settings where hard, metal sensors cannot be used.

As the TBM experiments were performed without heating the skin this also opens up the possibility for the system to provide truly continuous transcutaneous blood gas measurements.

## Conflicts of interest

Anders Persson and Greger Thornell are both partners in Fourth State Systems AB which produces the OES system used in this study.

## Supplementary Material
